# Editorial: Metabolism of Cancer Cells and Immune Cells in the Tumor Microenvironment

**DOI:** 10.3389/fimmu.2018.03080

**Published:** 2018-12-21

**Authors:** Yongsheng Li, Bo Zhu

**Affiliations:** ^1^Clinical Medicine Research Center, Army Medical University, Chongqing, China; ^2^Institute of Cancer, Xinqiao Hospital, Army Medical University, Chongqing, China

**Keywords:** metabolism, immune cells, cancer, tumor microenvironement, immune editing

Immune editing orchestrates the tumor initiation and progression. The crosstalk between tumor cells and immune cells in the tumor microenvironment (TME) manipulates the development of tumors. Although the recent advancement of immunotherapy was encouraging, and countless patients have achieved significant benefits, some patients still do not respond to immunotherapy, due to the complexity and diversity of the TME. Exploring the underlying mechanisms of TME-driven tumorigenesis and progression are essential for developing potential precise approaches for cancer treatment.

Cells require energy to maintain their survival, and various metabolites are also bioactive. It has now been recognized that metabolism regulates the phenotype and biological function of cells. In the TME, tumor cells and immune cells reprogram their metabolic patterns to adapt to the hypoxic, acidic, and low-nutrition microenvironment. For example, tumor cells display enhanced aerobic glycolysis but reduced oxidative phosphorylation (OXPHOS). Macrophages tend to be M2 polarized, exhibit upregulated fatty acid synthesis and β-oxidation. Cytotoxic T lymphocytes show dampened glycolysis but enhanced OXPHOS. Therefore, the metabolic reprogramming of various cells in the tumor microenvironment is bound to be of great significance for tumor immune editing. Understanding the metabolic reprogramming of tumor cells and immune cells will provide a new direction for regulating tumor immunity.

In this context, the goal of this research topic was to bring together a collection of thoughtful papers that review the advancement and prospect of the metabolism in cancer cells and immune cells and to inspire the researchers for future studies on the tumor immunity and metabolism, as well as to provide clues for clinical cancer therapy.

Hypoxia contributes to oncogenes activation and loss of tumor suppressors that constitute major regulators of Warburg effect and many other metabolic pathways such as glutaminolysis. The hypoxia-inducible factors promote angiogenesis via increasing vascular endothelial growth factors and modulate the cell phenotypes in the TME. Sormendi and Wielockx summarized the current knowledge of hypoxia-reprogrammed metabolism during cancer development and the mechanisms in cancer cells and immune cells in the TME. Endothelial cells (ECs) conduit for oxygen and nutrient delivery to tumor tissues. Zecchin et al. discussed how the ECs adapt their metabolism to form vessels in the TME.

Immunity and mitochondria are closely interlinked with each other. The mitochondria are the most important organelles for cell energy metabolism. They regulate activation, differentiation, and survival of immune cells, as well as release signals such as mitochondrial DNA (mtDNA) and mitochondrial ROS (mtROS) to regulate the transcription in immune cells. Angajala et al. discussed the underlying mechanism by which mitochondria coordinate to drive distinct immune responses.

Mevalonate metabolism is always fueled by glycolysis. It is a critical pathway for cancer stem cells and immune cells and governs immune surveillance. Gruenbacher and Thurnher discussed how activation and differentiation-induced metabolic reprogramming affects the mevalonate pathway for cholesterol biosynthesis in immune and cancer cells. They concluded that while inhibition of mevalonate metabolism in tumor cells may attenuate growth and proliferation, mevalonate pathway in innate immune cells such as macrophages may contribute to trained immunity.

The aryl hydrocarbon receptor (AhR) is an important cytosolic, ligand-dependent transcription factor and plays critical roles in the initiation, promotion, progression, invasion, and metastasis of cancer. Interestingly, a correlation between AhR and immune system has been recognized and suggested as an immunosuppressive effector. Xue et al. reviewed the role of AhR in tumor immunity and its potential mechanism in the TME.

T cells are major components for anti-tumor immunity. Their dynamic program of metabolism determines the differentiation, activation, and function. Manipulating the reprogramming of T-cell metabolic pathways is a therapeutic approach, in particular, for antitumor immunity. Kouidhi et al. illustrated some potential cell metabolism pathways involved in shaping T lymphocyte function and differentiation. They also demonstrated subsets of T cells have specific metabolic requirements and signaling pathways that contribute to their respective function.

In summary, the eight articles composing this research topic provide insights into key and complementary mechanisms underlying metabolism in cancer cells and immune cells in the TME. This issue will inspire researchers to explore questions on metabolic immunology and be beneficial for developing efficient strategies in clinical cancer therapy.

## Author Contributions

YL wrote the manuscript. BZ contributed to the discussion.

### Conflict of Interest Statement

The authors declare that the research was conducted in the absence of any commercial or financial relationships that could be construed as a potential conflict of interest.

